# Assessing Factors Associated with TB Awareness in Nepal: A National and Subnational Study

**DOI:** 10.3390/ijerph18105124

**Published:** 2021-05-12

**Authors:** Yoko Iwaki, Santosh Kumar Rauniyar, Shuhei Nomura, Michael C. Huang

**Affiliations:** 1 Science, Technology and Innovation Policy Program, National Graduate Institute for Policy Studies (GRIPS), 7-22-1 Roppongi, Minato-ku, Tokyo 106-8677, Japan; 2 Department of Global Health Policy, Graduate School of Medicine, The University of Tokyo, 7-3-1 Hongo, Bunkyo-ku, Tokyo 113-0033, Japan; rauniyar.sam@gmail.com (S.K.R.); nom3.shu@gmail.com (S.N.); 3 Department of Health Policy and Management, School of Medicine, Keio University, 35 Shinanomachi, Shinjuku-ku, Tokyo 160-8582, Japan; 4 SciREX Center, National Graduate Institute for Policy Studies (GRIPS), 7-22-1 Roppongi, Minato-ku, Tokyo 106-8677, Japan; c-huang@grips.ac.jp

**Keywords:** awareness of tuberculosis, TB symptoms, TB knowledge

## Abstract

Tuberculosis (TB) has still remained a serious global health threat in low- and middle-income countries in recent years. As of 2021, Nepal is one of the high TB burden countries, with an increasing prevalence of cases. This study evaluates factors associated with TB awareness in Nepal. This study uses data from the Nepal Demographic and Health Survey, a cross-sectional survey carried out from June 2016 to January 2017. Multilevel logistic regression is performed to examine the association of demographic and socioeconomic factors with TB awareness. Our findings show a high level of TB awareness in all seven provinces of Nepal. Province 5 has the highest level of awareness (98.1%) among all provinces, followed by provinces 3 and 4, while province 6 has the lowest awareness level (93.2%) compared to others. Socioeconomic factors such as wealth, education and owning a mobile phone are significantly associated with TB awareness. Socioeconomic determinants are influential factors associated with TB awareness in Nepal. The wide variation in the proportion of awareness at a regional level emphasizes the importance of formulating tailored strategies to increase TB awareness. For instance, the use of mobile phones could be an effective strategy to promote TB awareness at a regional level. This study provides valuable evidence to support further research on the contribution of information and communication technology (ICT) usage to improving TB awareness in Nepal.

## 1. Introduction

Tuberculosis (TB) has long been considered a critical infectious disease [1]. In 2019, 10 million people developed TB, leading to 1.4 million TB deaths worldwide [2]. The WHO Global TB Report 2020 found that 98% of the world’s registered TB cases were in low- and middle-income countries (LMICs) [2]. In LMICs, low awareness of TB is one of the leading risk factors for high prevalence [3]. Lack of TB awareness has become a severe social concern affecting population health, given the risk of spread of TB [4]. Inadequate knowledge about TB leads to further transmission and delay in diagnoses and treatment [5]. There are 3.6 million people with TB who are undiagnosed and hence do not receive treatment from healthcare facilities in LMICs [6]. As can be seen in the case of India, due to low TB awareness, diagnosis and treatment of TB have been delayed, leading to an increased probability of further spread of TB in the community [7].

In 1965, the national TB program (NTP) of Nepal was implemented by the Nepalese government in collaboration with the WHO and the United Nations International Children’s Emergency Fund (UNICEF). The main aim of the program is to effectively monitor and control TB incidence and prevalence and ensure better access to quality TB treatment services in Nepal [8]. Since 2006, almost 85% of TB cases in Nepal have been successfully treated through the NTP, and the incidence of TB has decreased gradually, by 3% per year until 2019 [9]. Despite efforts made by government and international organizations to end TB, around 40% of Nepalese participants in the *End TB* program did not seek TB care and treatment, according to Nepal’s 2018 national TB prevalence survey [9]. The results of the national survey indicate that there is inadequate knowledge of access to TB health services; thus, there is a need to increase awareness of the availability of quality TB diagnostics, care and treatment [6,8]. National health policy priorities include: achievement of the main goal, TB elimination; reducing the numbers of new TB cases and TB deaths (by enhancing TB awareness among the entire Nepalese population); removal of barriers to equitable access to TB treatment and care, to improve community wellbeing in regional Nepal [10]. All of those targets were set by the NTP at the central level and at local levels as well, in accordance with the goal of the national strategic plan, to *End TB* by 2030 in Nepal [8,10].

The level of TB awareness is associated with various demographic and socioeconomic factors such as education level, socioeconomic status and area of residence [8,11]. Low public awareness of and knowledge about TB have been identified as correlated with several socioeconomic factors including family income, education level and gender [12]. In addition, enhanced awareness of TB could improve access to appropriate treatment and better outcomes, especially in socioeconomically vulnerable groups—who have limited access to information about TB due to weak community engagement—and certain areas of residence. [13,14]. In LMICs, including Nepal, specific locations and places at greater distances from TB treatment facilities are susceptible to poor community TB awareness and weak adherence to TB treatment as a health-seeking behavior [15,16]. Examining the demographic and socioeconomic factors associated with TB awareness is important for understanding the socioeconomic gaps that can help to drive infectious TB control in the work to eliminate TB [12,17]. However, few studies have analyzed the magnitude of the associated factors at national and subnational levels in Nepal. Hence, the aim of our study is to evaluate the relationship between TB awareness and demographic and socioeconomic factors at national and subnational levels in Nepal.

## 2. Materials and Methods

### 2.1. Data-In-Use

In our study, we used data from the Nepal Demographic & Health Survey (NDHS) 2016. The NDHS is a nationally representative population-based cross-sectional household survey that obtains socioeconomic and disease-specific questionnaire information, including knowledge, attitudes and behaviors related to TB. The data was collected from June 2016 to January 2017. The overall response rate of the survey was 98.5%. We studied the data of individual household questionnaire responses for respondents aged 15–49 years. Privacy of household members was thoroughly ensured [18].

### 2.2. Outcome Variables and Covariates

Awareness of TB contributes to enhancing understanding of screening necessity, knowledge of good access to health facilities, completion of treatment as per programmatic management, and monitoring and supervision via community engagement; these factors have effective impacts on reducing TB cases [19,20]. The outcome variable included in our study of TB awareness was defined as people knowing about TB symptoms and having knowledge of TB treatment facilities. We considered access to healthcare services as a control variable in the analysis. The covariates included in our study were wealth quantiles, education, place of residence (urban and rural), province and owning a mobile phone. We selected the covariates based on previous empirical studies conducted in similar settings [12,21].

### 2.3. Statistical Analysis

We used descriptive statistics to analyze respondents’ demographic and socioeconomic characteristics. A multilevel regression model was used to explain the relationship between outcome variables and covariates. The data from the NDHS are nested in multiple levels, i.e., hierarchical in nature. Therefore, we used a multilevel regression model with a random intercept at a cluster level to account for the cluster-level effect in our analysis. The selection of covariates for the multilevel model was based on the significant results obtained from the univariate regression analysis. Both descriptive and multilevel regression analyses were adjusted to the complex survey design using sample weighting. Data were analyzed using STATA 16 (StataCrop LLC, College Station, TX, USA) and R 4.0.3 (The R Foundation for Statistical Computing, Indianapolis, IN, USA).

## 3. Results

### 3.1. Summary of Socio-Demographic Characteristics of Respondents

The summary statistics of the respondent’s characteristics are described in Table 1. We included 16,672 respondents (76.1% female and 23.9% male) aged between 15 and 49 years in our analyses. Among them, 96% reported awareness of TB. The proportion of participants aged 15–24 was 38.5%, while for 35–49 years of age, it was 31.6%. Looking at the education level of the respondents, around 50% of respondents had an education lower than secondary level, while 16.7% of respondents had a higher level of education. 76.8% of the total respondents owned a mobile phone. Finally, more than half (63.2%) of the respondents lived in an urban area.

### 3.2. Proportion of People with TB Awareness at a Subnational Level in Nepal

**Figure 1:**
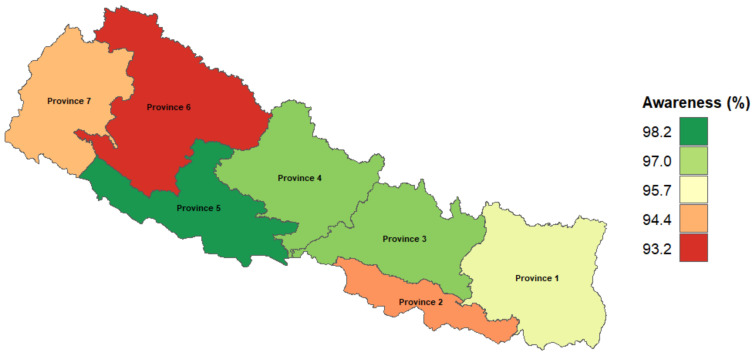
TB awareness at a subnational level in Nepal. Note: Provinces 1–7 represent the total number of provinces in Nepal. Province 1 = Not determined, Province 2 = Not determined, Province 3 = Bagmati, Province 4 = Gandagi, Province 5 = Lumbini, Province 6 = Karmali, Province 7 = Sudurpashchim.

The overall TB awareness at subnational levels in Nepal was above 90% across all provinces. Province 5 has the highest proportion of TB awareness (98.3%), followed by provinces 3 and 4 (97.4%); province 6 has the lowest awareness of all (93%) (Figure 1 & Appendix A Table A1).

### 3.3. Variables Associated with TB Awareness

The results of multilevel logistic regressions with a 95% confidence interval (CI), the odds ratio (OR) and respective *p*-values are presented in Table 2.

**Figure A1:**
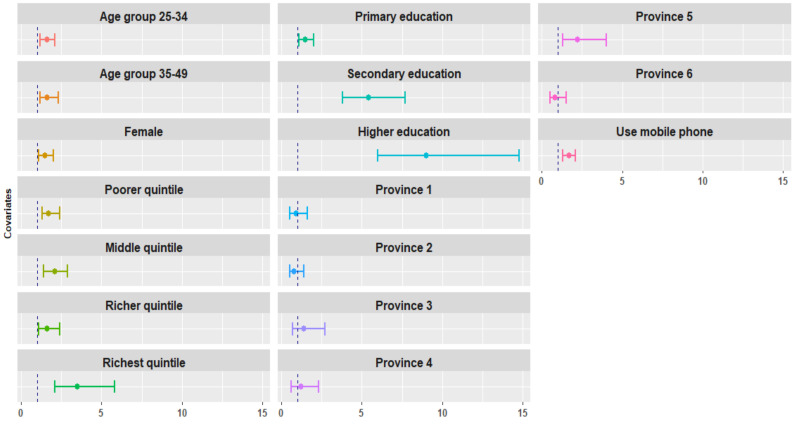
Factors associated with TB awareness. Note: Provinces 1–7 represent the total number of provinces in Nepal. Province 1 = Not determined, Province 2 = Not determined, Province 3 = Bagmati, Province 4 = Gandagi, Province 5 = Lumbini, Province 6 = Karmali, Province 7 = Sudurpashchim.

The odds of TB awareness among people in the richest quantile was 3.46 (95% CI 2.05–5.84) times higher compared to those in the poorest wealth quantile. In addition, the middle wealth quantile was also significantly related to TB awareness (OR 2.06, 95% CI 1.44–2.94). Respondents with higher education had significantly higher TB awareness (OR 16.19, 95% CI 8.04–32.58). Similarly, people with secondary level education had 5.36 (95% CI 3.76–7.65) times higher odds of being aware of TB compared to those with no formal education. Owning a mobile phone was significantly associated with TB awareness. Individuals owning a mobile phone had 1.66 (95% CI 1.30–2.10) times higher odds of being aware than those who did not own a mobile phone. Participants residing in province 5 were 2.24 (95% CI 1.26–3.97) times more likely to be aware of TB than those residing in province 6, which has the lowest proportion of TB awareness (Appendix B Figure A1).

## 4. Discussion

The prevalence of bacteriologically confirmed TB cases in Nepal increased from 374.5/100,000 population between 2003 and 2018 to 416.3 in 2019 [22,23]. It is likely that poor TB awareness delays treatment-seeking, resulting in TB prevalence increase, rather than other factors such as accessibility or economical constraints [24]. Hence, why to reduce the prevalence of TB, it is essential to increase TB awareness at national and subnational levels in Nepal. We examined the relationship between socioeconomic factors and awareness of TB at the national and subnational levels in Nepal. The results of our analysis indicated that wealth quintiles, education level, owning a mobile phone, and region of residence were significantly associated with higher TB awareness in Nepal.

People in the richest quintile have two times higher odds of being aware of TB compared to those in the poorest wealth quintile. This could be because the people in the richer and richest wealth quintiles tend to have better access to information and knowledge about TB symptoms than those in lower quintiles (the poorest and poorer). Similar results were obtained from the studies conducted in neighboring countries such as Pakistan and India [13,25]. In the case of Pakistan, it was found that more than half of households living in rural areas were of low socioeconomic status and had limited access to TB healthcare services [13]. Likewise, people of poor socioeconomic status in India—in particular, those living in poverty—were found to have a higher risk of an increased number of TB cases than those of higher socioeconomic status, partly because of lower access to quality healthcare services [25]. Nepal is a low-income country and 25% of its population lives below the poverty line [26,27]. People living in rural areas face a greater risk of delayed TB healthcare and treatment due to poor TB knowledge and limited access to basic health facilities [15,16].

Based on our findings, people who have completed secondary or higher education have more than five times higher odds of being well informed about TB compared to those without any formal education. This could be because people with secondary or higher education are more proactive about acquiring health information and are more concerned about their health than those without formal education [12,17]. According to some recent studies, people with a high education level know the symptoms of TB and recognize TB as a curable disease [11]. In addition, it has been found that there is a significant correlation between high school completion and TB knowledge in Africa [12]. Those findings support our result and suggest that a high socioeconomic status together with a high education level is strongly correlated with TB awareness and knowledge in LMICs.

People who own a mobile phone have 1.6 times higher odds of having awareness of TB compared to those who do not own a mobile phone. Due to increased affordability, mobile phone ownership has grown in LMICs [28]. It is estimated that around 81% of the total population of Nepal own mobile phones, which are now the most common information and communication devices [18]. Moreover, Nepalese mobile-cellular subscriptions per 100 inhabitants were estimated as 111.7 in 2017, the same level as in high-income countries [28]. Due to this access, there is considerable potential for the use of mobile phones in TB healthcare systems. Examining digital health and information and communication technology (ICT), Lester et al. [29] found that improvement of TB care adherence by using smartphones to communicate with patients may be an effective support for healthcare systems in LMICs. A case in point: in Cambodia, 97% of mobile phone users with TB can access TB health facilities in targeted intervention areas [30]. The growth of mobile phone use in relation to the health system offers a life-enhancing opportunity in LMICs, particularly for people of low socioeconomic status [31]. Planning better community engagement with digital communication tools for infection control is an important challenge [32], but it is necessary to understand the ICT for community-based adaption to health systems in LMICs because of resource limitations [33]. The utilization of smartphones in this context offers potential benefits such as supporting frequent communication and facilitating remote advice for patients, to provide faster, more accessible and more affordable TB healthcare information [2,8]. The diffusion of mobile phone usage in healthcare systems increases the need to understand that it is essential to enhance communication between healthcare providers and people with TB. Our findings indicate that using mobile phones to increase TB awareness could be an effective strategy to support a community-based healthcare approach to eliminating TB at the national and subnational levels in Nepal.

The region of residence variable was also significantly associated with the level of TB awareness. For instance, people living in province 5 are twice as likely to be aware of TB than those living in province 2, which has the lowest level of TB awareness among all provinces. Similarly, residents of province 3 have 1.5 times higher odds of being aware of TB than the residents of province 2. One of the reasons for that difference could be the fact that several TB programs have been implemented by international non-governmental organizations, such as the TB health support provided by the Japan International Cooperation Agency (JICA), starting from 1987. The final development phase by JICA was conducted from 2000 to 2005, focusing development assistance on provinces 3 (Kathmandu) and 5 (Rupandehi). Through a JICA assistance project, Japanese health experts provided technical training to medical workers to improve their healthcare skills at a Directly Observed Treatment Short-Courses (DOTs) center and sub-healthcare centers in provinces 3 and 5 [34]. JICA’s TB health assistance report found that a multi-dimensional approach to decrease the burden of TB has the potential to support quality TB healthcare services in the local communities in Nepal [35]. Several orientations and workshops were conducted, for medical staff and mother’s groups, in provinces 3 and 5 through JICA’s TB project [34]. Resulting changes in those people’s behavior toward TB are supportive of a holistic and community-centered approach in healthcare systems led by workshops. It is proposed that people aware of TB who are living in provinces 3 and 5 could facilitate others’ TB awareness for several years. Further development assistance projects may also be conducted to maintain TB health at a community level; this needs to be factored into the complex situation. Another reason for greater awareness in province 3 could be the fact that it is urbanized. The capital city of Nepal is located in province 3, along with many other cities with a high number of healthcare facilities. Indeed, patients with TB in province 3 have the highest TB treatment success rate (94%) thanks to the high number of healthcare facilities, including TB treatment centers, DOTs centers and TB laboratories, compared to other provinces [23]. From the above, it is evident that living in an urban area is one of the factors associated with TB awareness in Nepal.

This research faced the inevitable problem related to household survey analysis: participants whose information was not complete were excluded from the study. Most importantly, people aged 49 and over were excluded from our study because of data limitations. This exclusion could have led to an overestimation of the TB-aware population, if older citizens were less likely to be aware of TB. Secondly, TB awareness could be influenced by several factors including those related to distance from healthcare facilities, attitude of service providers, and disease complications. However, due to limited data availability, we could not include and analyze those factors. Finally, several TB awareness programs were conducted in different regions of Nepal, which could have affected regional awareness. A thorough analysis of TB programs could enhance the findings of this study.

## 5. Conclusions

Our study examined the demographic and socioeconomic factors associated with TB awareness at the national and subnational levels in Nepal. The results of the study highlighted that socioeconomic determinants such as wealth quintile, level of education, and owning a mobile phone were significantly associated with TB awareness at the national and regional levels. The high level of awareness at a regional level emphasizes the importance of formulating tailored strategies to increase TB awareness. The use of mobile phones could be an effective strategy to promote TB awareness at a regional level. The policy implementation of a mobile-focused approach for medical infrastructure could improve TB awareness and access to treatment, effectively and affordably. This study provides valuable evidence to support further research on the contribution of ICT usage to improving TB awareness in Nepal. Further research is required to understand the possible mechanisms that affect the underlying factors determining TB awareness in the context of Nepal, to help to promote and support TB elimination in Nepal.

## Figures and Tables

**Table 1:** Demographic and socioeconomic characteristics of study participants, 2016.

Characteristics	Number	Proportion (%)
Total	16,672	100
Gender		
Male	3978	23.9
Female	12,694	76.1
Age group		
15–24	6424	38.5
25–34	4991	29.9
35–49	5257	31.6
Educational attainment		
No formal education	4534	27.2
Primary education	2889	17.3
Secondary education	6459	38.8
Higher education	2790	16.7
Province		
Province 1 (Not determined)	2826	17.0
Province 2 (Not determined)	3323	19.9
Province 3 (Bagmati)	3670	22.0
Province 4 (Gandagi)	1604	9.6
Province 5 (Lumbini)	2886	17.3
Province 6 (Karmali)	915	5.5
Province 7 (Sudurpashchim)	1448	8.7
Owns a mobile phone		
No	3861	23.2
Yes	12,811	76.8
Residence		
Urban	10,543	63.2
Rural	6129	36.8

**Table 2:** Demographic and socioeconomic factors associated with TB awareness in Nepal, 2016.

Variables	TB Awareness Odds Ratio (95% CI)
Age group	
15–24	1.00 (ref)
25–35	1.57 (1.19–2.08) **
35–49	1.61 (1.15–2.26) **
Gender	
Male	1.00 (ref)
Female	1.46 (1.05–2.04) *
Wealth quintile	
Poorest	1.00 (ref)
Poorer	1.74 (1.25–2.42) **
Middle	2.06 (1.44–2.94) ***
Richer	1.64 (1.14–2.36) **
Richest	3.46 (2.05–5.84) ***
Education	
No formal education	1.00 (ref)
Primary education	1.48 (1.10–1.99) *
Secondary education	5.36 (3.76–7.65) ***
Higher education	16.19 (8.04–32.58) ***
Owns a mobile phone	
No	1.00 (ref)
Yes	1.66 (1.30–2.10) ***
Province	
Province 6 (Karmali)	1.00 (ref)
Province 1 (Not determined)	0.87 (0.49–1.57)
Province 2 (Not determined)	0.79 (0.45–1.41)
Province 3 (Bagmati)	1.42 (0.74–2.71)
Province 4 (Gandagi)	1.21 (0.63–2.31)
Province 5 (Lumbini)	2.24 (1.26–3.97) *
Province 7 (Sudurpashchim)	0.84 (0.48–1.50)

TB: Tuberculosis. * *p* < 0.05; ** *p* < 0.01; *** *p* < 0.001.

## Data Availability

The datasets analyzed during the study are available in the Demographic and Health Surveys of the DHS repository, https://dhsprogram.com/data/dataset/Nepal_Standard-DHS_2016.cfm?flag=0 (accessed on 3 February 2021).

## Appendix A

**Table A1:** People with TB awareness at a subnational level in Nepal.

	Proportion (95% Confidence Interval)
	TB Awareness
Province 1 (Not determined)	96.1 (95.3–96.7)
Province 2 (Not determined)	94.1 (93.3–94.9)
Province 3 (Bagmati)	97.4 (96.9–97.9)
Province 4 (Gandagi)	97.4 (96.5–98.1)
Province 5 (Lumbini)	98.1 (97.6–98.6)
Province 6 (Karmali)	93.2 (91.3–94.6)
Province 7 (Sudurpashchim)	94.5 (93.2–95.6)

TB: Tuberculosis.
